# Morphological investigations of endomorphin-2 and spinoparabrachial projection neurons in the spinal dorsal horn of the rat

**DOI:** 10.3389/fnana.2022.1072704

**Published:** 2022-11-23

**Authors:** Jun-Bin Yin, Ya-Cheng Lu, Fei Li, Ting Zhang, Tan Ding, Huai-Qiang Hu, Ying-Biao Chen, Hong-Wei Guo, Zhen-Zhen Kou, Ming-Ming Zhang, Jun Yuan, Tao Chen, Hui Li, Bing-Zhen Cao, Yu-Lin Dong, Yun-Qing Li

**Affiliations:** ^1^Department of Human Anatomy, K. K. Leung Brain Research Centre, The Fourth Military Medical University, Xi’an, China; ^2^Department of Neurology, The 960th Hospital of Joint Logistics Support, PLA, Jinan, China; ^3^State Key Laboratory of Military Medical Psychology, The Fourth Military Medical University, Xi’an, China; ^4^Institute of Orthopedics, Xijing Hospital, The Fourth Military Medical University, Xi’an, China; ^5^Department of Human Anatomy, Fujian Health College, Fuzhou, China

**Keywords:** endomorphin-2, spinal dorsal horn, projection neurons, μ-opioid receptor, presynaptic effects

## Abstract

It has been proved that endomorphin-2 (EM2) produced obvious analgesic effects in the spinal dorsal horn (SDH), which existed in our human bodies with remarkable affinity and selectivity for the μ-opioid receptor (MOR). Our previous study has demonstrated that EM2 made synapses with the spinoparabrachial projection neurons (PNs) in the SDH and inhibited their activities by reducing presynaptic glutamate release. However, the morphological features of EM2 and the spinoparabrachial PNs in the SDH have not been completely investigated. Here, we examined the morphological features of EM2 and the spinoparabrachial PNs by using triple fluorescence and electron microscopic immunohistochemistry. EM2-immunoreactive (-ir) afferents directly contacted with the spinoparabrachial PNs in lamina I of the SDH. Immunoelectron microscopy (IEM) were used to confirm that these contacts were synaptic connections. It was also observed that EM2-ir axon terminals contacting with spinoparabrachial PNs in lamina I contained MOR, substance P (SP) and vesicular glutamate transporter 2 (VGLUT2). In lamina II, MOR-ir neurons were observed to receive direct contacts from EM2-ir varicosities. The synaptic connections among EM2, MOR, SP, VGLUT2, and the spinoparabrachial PNs were also confirmed by IEM. In sum, our results supply morphological evidences for the analgesic effects of EM2 on the spinoparabrachial PNs in the SDH.

## Introduction

Endomorphin (EM) had remarkable affinity and selectivity for the μ-opioid receptor (MOR), which included EM1 (Tyr-Pro-Trp-Phe-NH2) and EM2 (Tyr-Pro-Phe-Phe-NH2) (Zadina et al., 1997; Gu et al., 2017). It was considered that EM1 played an important role in brain nucleus such as the periaqueductal gray (Chen et al., 2016), while EM2 mainly functioned in the spinal dorsal horn (SDH) (Tseng et al., 2000; Yin et al., 2018; Lu et al., 2022). EM2 plays a pivotal role in inhibiting nociceptive information transmission at the spinal cord level (Stone et al., 1997; Zadina et al., 1997). EM2-immunoreactive (-ir) fibers existed in the superficial lamina of the SDH (Yin et al., 2018; Lu et al., 2022), which was released following the dorsal root entry zone receiving electrical stimulation (Williams et al., 1999; Heinke et al., 2011). Intrathecal (*i.t.*) administrations of EM2 produced obvious analgesic effects in the paw-withdrawal, tail pressure, tail-flick and flexor-reflex tests on rodents (Stone et al., 1997; Ohsawa et al., 2001; Grass et al., 2002; Chen et al., 2015; Zhao et al., 2015).

Previously, the underling mechanisms for these analgesic effects of EM2 in the SDH mainly focused on the investigations of interneurons in the substantia gelatinosa (SG). MOR-ir neurons received contacts from EM2-containing axons in the SG, which also contained substance P (SP) (Spike et al., 2002). Most of these EM2-containing axon terminals made asymmetrical synapses with MOR-ir profiles (Wang et al., 2003). Membrane potentials of neurons in the SG were hyperpolarized by EM2, which was mediated by MOR activation opening inwardly-rectifying K + channels (Wu et al., 1999, 2003; Fujita and Kumamoto, 2006). However, in all of these reports, the projection neurons (PNs) in the SDH have rarely been investigated, and the effects of EM2 on the PNs remained obscure. There are various interneurons and local neural circuits for transmitting, coding, and integrating the nociceptive information in the SDH. No matter how many interneurons and local neural circuits were involved in processing the nociceptive information, the PNs are the last station transmitting the signals output from the SDH. Therefore, PNs have critical roles in relaying primary afferent information directly to nociceptive brain centers (Marshall et al., 1996). Recent evidences also indicate that the PNs may play key roles in the development and maintenance of chronic pain (Basbaum et al., 2009; Todd, 2010). Thus, the morphological features of the PNs in lamina I are important for us to understand how the nociceptive information were transmitted to the brain and how the central sensitization for the chronic pain was induced. Our previous study has shown that EM2-ir axons directly formed synapses with the spinoparabrachial PNs and applying EM2 inhibited the activities of these PNs through reducing presynaptic release of glutamate from the primary afferent terminals (Yin et al., 2018).

In this study, we will present the morphological evidences for the analgesic effects of EM2 on the spinoparabrachial PNs in the SDH by using triple fluorescence and electron microscopic immunohistochemistry. The distributions of EM2, MOR, and the spinoparabrachial PNs in the SDH will be checked. The co-expressions of EM2 and SP, or VGLUT2, or GAD67 will also be investigated in the SDH. The ultra-structures of these neurotransmitters will be examined by using immunoelectron microscopy.

## Materials and methods

### Animals

Adult male Sprague-Dawley (SD) rats (weighting 250–300 g) were housed in a temperature-controlled environment, with food and water *ad arbitrium*. The Animal Center of the Fourth Military Medical University (Xi’an, China) supplied all these rats. All procedures of our experiments were carried out in accordance with the International Association for the Study of Pain guidelines (Zimmermann, 1983) for the care and using of laboratory animals, which were also approved by the Committee of Animal Use for Research and Education of the Fourth Military Medical University.

### Stereotaxic microinjections

Stereotaxic microinjections followed the procedures described in our previous studies (Yin et al., 2014, 2018). All rats were anesthetized with intraperitoneal (*i.p.*) injection of 2% sodium pentobarbital (40 mg/kg). A hole was made on the skull with a dental drill to insert a microsyringe (1 μl, Hamilton, Reno, NV, USA) into the lateral parabrachial nucleus (LPB), according to the coordinates: 9.0 mm caudal to Bregma, 2.3 mm lateral to the midline and 7.0 mm ventral to the surface of the cranium (Paxinos and Watson, 2005). A volume of 0.04 μl of 4% (w/v) Fluoro-Gold (FG; Fluorochrome; 80014; Biotium; Hayward, CA, USA) was pressure-injected into the LPB. Similarly, 0.1 μl of 2% WGA-HRP (Toyobo, Osaka, Japan) was also injected into the LPB with the above coordinates. Each microinjection proceeded slowly and the needle was kept in the LPB for 10 min post injection. After the stereotaxic microinjections, the rats injected with FG were allowed to survive for 7 d. But, the rats injected with WGA-HRP were only allowed to survive for 60 h.

### Immunofluorescence histochemical multi-staining

Six rats injected with FG were anesthetized with *i.p.* injection of 2% sodium pentobarbital (100 mg/kg). Then, the rats were perfused with l00 ml of 0.9% saline, followed by 500 ml of 4% paraformaldehyde and 30% saturated picric acid in 0.1 M phosphate buffer (PB, pH 7.4). After the perfusions finished, both brains and lumbar spinal cord (SC) were taken immediately. These tissues were immersed in the same fixative for 4 h at 4°C, and then which were transferred to 30% sucrose in 0.1 M PB overnight at 4°C. After being embedded in an inert mounting medium (OCT; Tissue-Tek; Sakura; Torrance, CA, USA), a freezing microtome (Kryostat 1720; Leitz, Germany) were used to cut the brain and SC into coronal sections at 50 and 30 μm-thick, respectively. The sections containing LPB and lumbar SC were collected into eight dishes, which were filled with 0.01 M phosphate-buffered saline (PBS, pH 7.4). The sections in the first dish were mounted onto gelatin-coated glass slides, which were cover-slipped with a mixture of 50% glycerin and 2.5% triethylenediamine following air dried. A fluorescence microscope (Olympus BX-60; Tokyo, Japan) were used for observing the injection sites of FG in the LPB and the FG labeled projection neurons in the SC.

The procedures in immunofluorescence histochemical multi-staining were the same as those in our previous studies (Hui et al., 2010; Yin et al., 2014, 2018). The second to the seventh sets of the SC sections were adopted to examine the multi-staining of EM2/FG and triple-labeling of EM2/MOR/FG, EM2/MOR/NeuN, EM2/SP/FG, EM2/VGLUT2/FG, and EM2/GAD67/FG, respectively. Table 1 presented the antibodies used in the multi-stainings. These sections were blocked with 10% normal donkey serum (NDS) for 0.5 h, then subjected to the following incubations with: (1) primary antisera in the dilution medium [5% NDS, 0.3% Triton X-100, 0.05% NaN3 and 0.25% λ-carrageenan in 0.01 M PBS (PBS-NDS, pH 7.4)] overnight at room temperature (RT) and then 72 h at 4°C; (2) secondary antisera in PBS-NDS for 6 h at RT; (3) FITC-conjugated avidin (A-2001, Vector; Burlingame, CA, USA) in PBS containing 0.3% Triton X-100 (PBS-X, pH 7.4) for 3 h at RT. For EM2/MOR/FG and EM2/VGLUT2/FG triple staining, sections were re-incubated with rabbit antiserum against FG (AB153-I, Millipore; Billerica, MA, USA) overnight followed with Alexa647-donkey anti-rabbit IgG (A-31573, Invitrogen; Eugene, OR, USA) for 6 h at RT diluted with PBS-NDS, after EM2/MOR or EM2/VGLUT2 double staining using the above procedures (Liu et al., 2011; Baseer et al., 2012, 2014). The sections were washed completely with 0.01 M PBS between each step.

**TABLE 1:** Antisera used in each group for light microscopy.

Purposes	Primary antisera	Secondary antisera	Tertiary antisera
EM2/FG	Rabbit anti-EM2 (1:200)	Biotinylated donkey anti-rabbit (1:500)	FITC-Avidin (1:1,000)
	Guinea pig anti-FG (1:500)	Alexa 594 donkey anti-guinea pig (1:500)	
EM2/MOR/FG	Rabbit anti-EM2 (1:200)	Biotinylated donkey anti-rabbit (1:500)	FITC-Avidin (1:1,000)
	Guinea pig anti-MOR (1:500)	Alexa 594 donkey anti-guinea pig (1:500)	
	Rabbit anti-FG (1:1,000)	Alexa 647 donkey anti-rabbit (1:500)	
EM2/MOR/NeuN	Rabbit anti-EM2 (1:200)	Biotinylated donkey anti-rabbit (1:500)	FITC-Avidin (1:1,000)
	Guinea pig anti-MOR (1:500)	Alexa 594 donkey anti-guinea pig (1:500)	
	Mouse anti-NeuN (1:2,000)	Alexa 647 donkey anti-mouse (1:500)	
EM2/SP/FG	Rabbit anti-EM2 (1:200)	Biotinylated donkey anti-rabbit (1:500)	FITC-Avidin (1:1,000)
	Rat anti-SP (1:200)	Cy3 donkey anti-rat (1:500)	
	Guinea pig anti-FG (1:500)	Alexa 647 donkey anti-guinea pig (1:500)	
EM2/VGLUT2/FG	Rabbit anti-EM2 (1:200)	Biotinylated donkey anti-rabbit (1:500)	FITC-Avidin (1:1,000)
	Guinea pig anti-VGLUT2 (1:5,000)	Alexa 594 donkey anti-guinea pig (1:500)	
	Rabbit anti-FG (1:1,000)	Alexa 647 donkey anti-rabbit (1:500)	
EM2/GAD67/FG	Rabbit anti-EM2 (1:200)	Biotinylated donkey anti-rabbit (1:500)	FITC-Avidin (1:1,000)
	Mouse anti-GAD67 (1:500)	Alexa 594 donkey anti-rat (1:500)	
	Guinea pig anti-FG (1:500)	Alexa 647 donkey anti-guinea pig (1:500)	

The detailed information of antisera used in the current study were rabbit antiserum against EM2 (AB5104, Chemicon; Temecula, CA, USA), guinea pig antiserum against FG (NM-101, PROTOS BIOTECH CORP; New York, NY, USA), guinea pig antiserum against MOR1 (AB1774, Millipore), mouse antiserum against NeuN (MAB377, Millipore), rat antiserum against SP (MAB356, Millipore), guinea pig antiserum against VGLUT2 (AB2251, Millipore), mouse antiserum against GAD67 (MAB5406, Millipore), rabbit antiserum against FG (AB153-1, Millipore), biotinylated donkey anti-rabbit IgG (AP182F, Millipore), Alexa594-donkey anti-guinea pig IgG (706-585-148, Jackson ImmunoResearch; Suffolk, UK), Alexa647-donkey anti-mouse IgG (A-31571, Invitrogen), Cy3-donkey anti-rat IgG (AP189c, Millipore), Alexa647-donkey anti-guinea pig IgG (AP193SA6, Millipore), Alexa594-donkey anti-mouse IgG (A-21203, Invitrogen).

The eighth set of sections was used as control. The primary antisera were replaced with normal serum and the other procedures followed the above groups. After the staining was finished, all sections were mounted onto glass slides. The laser scanning confocal microscopy (FV1000, Olympus, Japan) was used to observe the results, under appropriate filters for FITC (excitation 492 nm; emission 520 nm), Alexa594 (excitation 590 nm; emission 618 nm), and Alexa647 (excitation 647 nm; emission 666 nm). For EM2-ir boutons contacting the MOR-ir neurons, the surface areas of these neurons were measured. The surface areas of dendrites were also estimated based on their lengths and diameters (Baseer et al., 2012, 2014).

### Immunoelectron microscopy

Six rats injected with WGA-HRP were anesthetized with *i.p.* injection of 2% sodium pentobarbital (100 mg/kg). Then, the rats were perfused with l00 ml of 0.9% saline, followed by 500 ml of 4% paraformaldehyde, 0.1% glutaraldehyde, and 15% saturated picric acid in 0.1 M PB. After the brains and SC were quickly removed, a vibratome (Microslicer DTM-1000, DSK, Kyoto, Japan) was used to cut the pons and lumbar SC into 50 μm-thick coronal sections. The sections were collected into 5 dishes containing 0.1 M PB. Then, the WGA-HRP was examined by using the tetramethylbenzidine-sodium tungstate (TMB-ST) method (Gu et al., 1992; Wang et al., 2014; Yin et al., 2018). A DAB/cobalt/H_2_O_2_ solution was used to intensify the WGA-HRP reaction products (Rye et al., 1984). The SC sections were examined under a light microscope and those containing WGA-HRP-labeled neurons in the lamina I were selected. Vials were used to collect these selected sections, which contained a mixture of 25% sucrose and 10% glycerol in 0.05 M PB. Then, the vials were treated in liquid nitrogen for enhancing the penetrations of antibodies in the following reactions (Chen et al., 2016; Yin et al., 2018). At last, the sections were washed three times in 0.05 M Tris-buffered saline (TBS; pH 7.4) and then blocked with 0.05 M TBS containing 20% NDS for 30 min.

The procedures for staining of EM2-, MOR-, SP-, VGLUT2-, and GAD67-ir profiles were the same as our previous studies (Yin et al., 2018; Lu et al., 2022). All of the antisera used are presented in Table 2. The sections in the first dish were used for EM2/HRP double staining. Rabbit antiserum against EM2 (1:100) was used to incubate the sections for 24 h at RT then for 72 h at 4°C, which was diluted with 2% NDS in 0.05 M TBS (pH 7.4) (TBS-NDS). Then, these sections were incubated in biotinylated donkey anti-rabbit IgG (1:200) overnight and finally with the ABC kit (1:100; Vector) for 4 h. After incubations, 0.05 M Tris-HCl (pH 7.6) containing 0.02% DAB and 0.003% H_2_O_2_ was used to proceed the sections for 10–20 min.

**TABLE 2:** Antisera used in each group for electron microscopy.

Purposes	Primary antisera	Secondary antisera	Tertiary antisera
EM2/HRP*	Rabbit anti-EM2 (1:100)	Biotinylated donkey anti-rabbit (1:200)	ABC Elite Kit (1:100)
EM2/MOR/HRP*	Rabbit anti-EM2 (1:100)	Biotinylated donkey anti-rabbit (1:200)	ABC Elite Kit (1:100)
	Guinea pig anti-MOR (1:200)	Goat anti-guinea pig IgG antibody conjugated to 1.4 nm gold particles (1:100)	
EM2/SP/HRP*	Rabbit anti-EM2 (1:100)	Biotinylated donkey anti-rabbit (1:200)	ABC Elite Kit (1:100)
	Rat anti-SP (1:100)	Goat anti-rat IgG antibody conjugated to 1.4 nm gold particles (1:100)	
EM2/VGLUT2/HRP*	Rabbit anti-EM2 (1:100)	Biotinylated donkey anti-rabbit (1:200)	ABC Elite Kit (1:100)
	Guinea pig anti-VGLUT2 (1:1,000)	Goat anti-guinea pig IgG antibody conjugated to 1.4 nm gold particles (1:100)	
EM2/GAD67/HRP*	Rabbit anti-EM2 (1:100)	Biotinylated donkey anti-rabbit (1:200)	ABC Elite Kit (1:100)
	Mouse anti-GAD67 (1:200)	Goat anti-mouse IgG antibody conjugated to 1.4 nm gold particles (1:100)	

*The DAB reaction utilized for the demonstration of HRP in each group was performed before the immuno-electron microscopy procedures.

The sections in the other dishes were incubated with: (1) primary antisera in the TBS-NDS for 24 h at RT then for 72 h at 4°C; (2) secondary antisera in TBS-NDS for overnight at RT. The detailed information of antisera used were goat anti-guinea pig IgG conjugated to 1.4-nm gold particles (20541, Nanoprobes; Yaphank, NY, USA), goat anti-rat IgG conjugated to 1.4-nm gold particles (2007, Nanoprobes), and goat anti-mouse IgG conjugated to 1.4-nm gold particles (2001, Nanoprobes). Subsequently, all sections were incubated in the following solutions: (1) post-fixation with glutaraldehyde, (2) the HQ Silver Kit (Nanoprobes) for silver enhancement, (3) the ABC kit, (4) DAB tetrahydrochloride and H_2_O_2_. 1% solution of osmium tetroxide was used to post-fix the above sections for 45 min. And then the sections were stained with 1% uranyl acetate in 70% ethanol for 1 h. Subsequently, an Eposy resin (Fluka Chemie, Buchs, Switzerland) was used to embed the sections following dehydration in a graded series of ethanol and degrease in propylene oxide. Under a dissection microscope, pieces of SDH areas containing WGA-HRP-labeled neurons were cut out from the whole sections with fresh razor blades. Then, the pieces were cut into 70-nm thick ultrathin sections with a diamond knife mounted on an ultramicrotome (Reichert-Nissei Ultracut S; Leica, Wein, Austria). The series of ultrathin sections were mounted on single-slot grids coated with pioloform membrane (Agar Scientific, Stansted, UK), and then stained with 1% (w/v) lead citrate. Lastly, an electron microscope (JEM1440, Tokyo, Japan) was used to observe the ultrastructures in the SDH.

## Results

### Endomorphin-2-immunoreactive (ir) fibers and terminals contact spinoparabrachial projection neurons in lamina I

**FIGURE 1:**
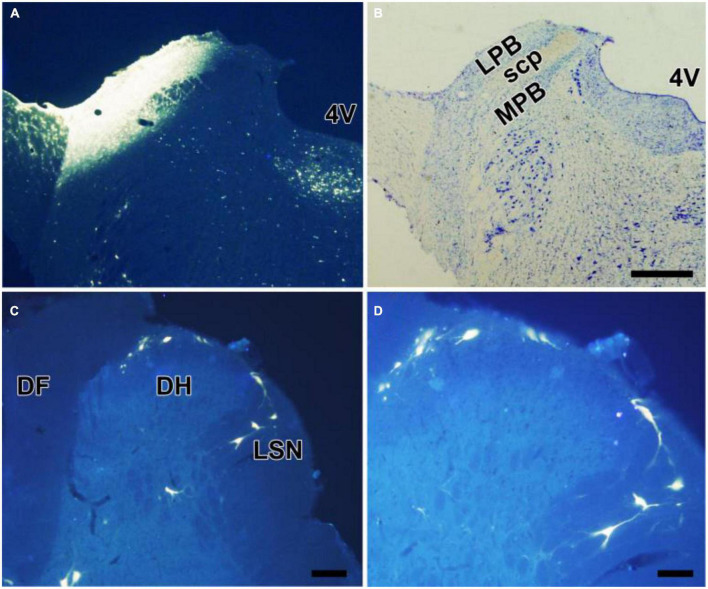
Retrograde labeling of neurons projecting to the LPB in the spinal cord. **(A,B)** FG was injected into the LPB on the left side **(A)**. The cytoarchitectonic areas involved in the injection site were confirmed by cresyl violet counterstaining of the same section **(B)**. Some neurons in lamina I, III, and V, presumably projection neurons, were labeled with FG **(C,D)**. 4V, fourth ventricle; LPB, laterial parabrachial nucleus; MPB, medial parabrachial nucleus; SCP, superior cerebellar peduncle. Scale bars = 200 μm (in **B** also for **A**), 100 μm (in **C**), and 50 μm (in **D**).

**FIGURE 2:**
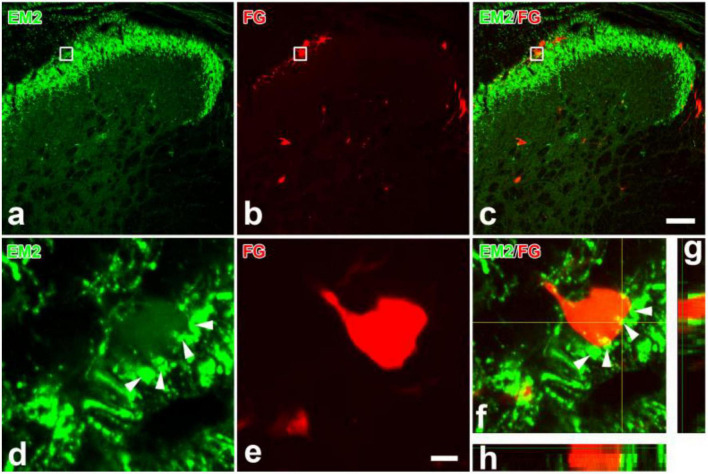
Immunofluorescent histochemical double-staining showing the connections between EM2-ir terminals (**a,c,d,f–h**, green) and FG labeled spinoparabrachial projection neurons (**b**,**c**, **e–h**, red) in lamina I. The rectangle areas in **(a–c)** were enlarged in **(d–f)**, respectively. The merged 3D images in **(f–h)** revealed close contacts between EM2-ir terminals (green) and one FG-labeled cell body (red). The arrowheads in **(d,f)** showed EM2-ir terminals connecting to the projection neurons. I–IV: lamina I to lamina IV. Scale bars = 100 μm (in **a–c**) and 8 μm (in **d,e**).

All rats presented with the FG injected into the LPB, with a few spread into nucleus adjacent to the LPB (Figures 1A,B). Lots of FG-labeled projection neurons were seen throughout the bilateral SDH. The spinoparabrachial PNs mostly distributed in lamina I, the deeper lamina of the SDH (III and V), and the lateral spinal nucleus, with a dominance on the SDH contralateral to the injection side (Figures 1C,D). EM2-ir fibers and terminals densely distributed in the lamina I and IIo, less in other lamina (Figure 2a). Many EM2-ir terminals closely connected with the spinoparabrachial PNs in lamina I (Figures 2b–h). There were no EM2-ir neuronal bodies observed in the SDH.

### Distributions of endomorphin-2-ir terminals and μ-opioid receptor-ir profiles in the superficial spinal dorsal horn

**FIGURE 3:**
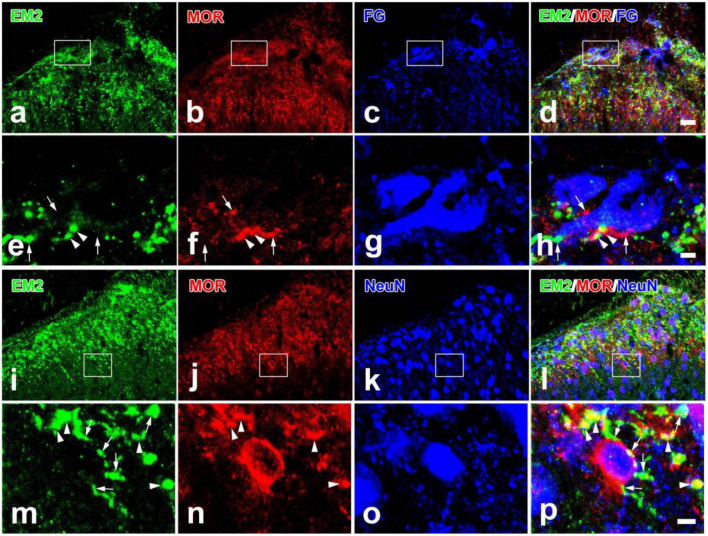
Immunofluorescent histochemical triple-staining showing the relationships among EM2-ir terminals (**a,d,e,h,i,l,m,p**, green), MOR-ir profiles (**b,d,f,h,j,l,n,p**, red) and neuronal cell bodies [FG-labeled projection neurons in **(c,d,g,h)**; NeuN-ir neurons in **(k,l,o,p)**; blue] in the spinal dorsal horn. The rectangle areas in **(a–d)** and **(i–l)** were enlarged in **(e–h)** and **(m–p)**, respectively. The merged image in **(h)** revealed close contacts between EM2/MOR-ir varicosities and FG-labeled projection neurons (arrowheads also in **e**,**f**) and contacts between EM2- or MOR-ir varicosities and projection neurons (arrows also in **e**,**f**). The merged image in **(p)** revealed close contacts between EM2-ir varicosities and MOR/NeuN-expressing neurons (arrows also in **m**) and varicosities containing both EM2- and MOR-ir (arrowheads also in **m,n**). Scale bars = 20 μm (in **a–d** and **i–l**), 4 μm (in **e–h**) and 4 μm (in **m–p**).

As the matching distributions of EM2 and MOR in the SDH, the relationship among EM2-ir terminals, MOR-ir profiles, and PNs in lamina I were investigated by using triple immunofluorescent staining. Moderate to intense MOR-ir terminals and neurons were observed in neuropil of laminae I and II (Figures 3b,j). Axonal varicosities showing immunopositive staining for both EM2 and MOR were observed to form contacts with some dendritic processes and cell bodies of the FG-labeled PNs (Figures 3a–h). Among the EM2-ir varicosities in contacting with FG-labeled PNs, the proportion of those were also MOR-ir was 58.84 ± 9.47% in the SDH. The PNs also received contacts from single-MOR-ir axons; however, these were much less. A number of small cell bodies with MOR-ir were seen in lamina II, whereas cell bodies expressing MOR were occasionally detected in lamina I and III (Figures 3b,f,j,n). As known to us, NeuN-ir cells presented throughout the gray matter in the SDH (Figures 3k,l). MOR-ir staining reaction products were located in the entire somatic plasma membrane (Figure 3n). From all rats adopted in the study, 2009 NeuN-ir neurons in lamina II were analyzed and 193 of these (9.6%) were MOR-ir. In lamina II, EM2-ir varicosities contacting MOR-ir interneurons can be seen (Figures 3i–l, m–p). The density of these contacts on membranes of analyzed 21 MOR-ir interneurons varied from 1.89 to 8.53/100 μm^2^ (mean 4.49 ± 0.49 SEM).

### The relationships between endomorphin-2- and substance P- or vesicular glutamate transporter 2- or GAD67-ir profiles in the superficial spinal dorsal horn

**FIGURE 4:**
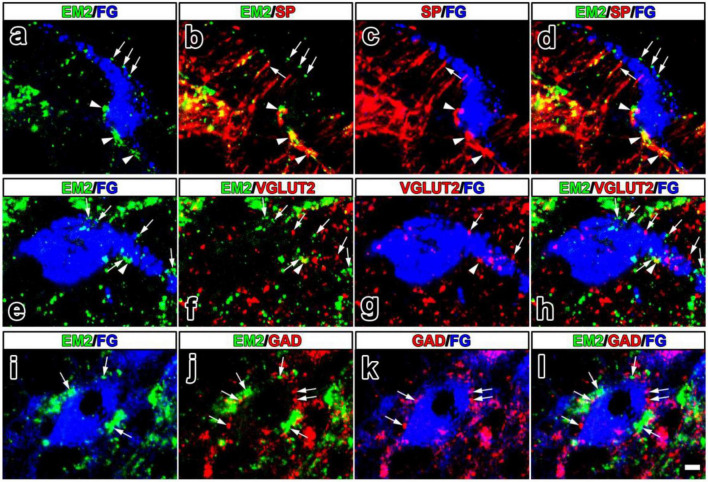
The fluorescence photomicrographic images of triple-labeling showing the contacts between EM2-ir primary afferents and/or SP-, VGLUT2-, GAD67-ir varicosities and FG-labeled projection neurons. The merged image in **(d)** revealed close contacts between varicosities containing both EM2 (green) and SP (red) and FG-labeled projection neurons (blue) (arrowheads also in **a–c**). Varicosities containing either EM2 or SP also contacted FG-labeled projection neurons (arrows in **a–d**). The merged image in **(h)** reveals close contacts between varicosities containing both EM2 (green) and VGLUT2 (red) and FG-labeled projection neurons (blue) (arrowheads also in **e–g**). Varicosities containing either EM2 or VGLUT2 also contacted FG-labeled projection neurons (arrows in **e**–**h**). The merged image in **(l)** revealed close contacts between varicosities containing either EM2 (green) or GAD67 (red) and FG-labeled projection neurons (blue) (arrows in **i–l**). Scale bar = 9 μm (in **a–l**).

Given the presynaptic distribution of EM2 and MOR to the PNs, we would like to check the properties of EM2-ir varicosities in contacting with PNs to better understand the presynaptic mechanism of its analgesic effects. In lamina I and II of the SDH, lots of SP-ir axons also presented EM2-ir (Figure 4b). The EM2-ir profiles were generally restricted to varicosities in these axons, whereas SP-ir profiles were often followed into inter-varicose segments. FG-labeled PNs in lamina I received all these above varicosities containing EM2 and/or SP (Figures 4a–d). Among the EM2-ir varicosities in contacting with FG-labeled PNs, the proportion of those were also SP-ir was 71.18 ± 7.25% in the SDH. VGLUT2-immunoreactivity was most intense in lamina I and lamina IIo. The FG-labeled PNs in lamina I also received contacts from varicosities containing EM2 and/or VGLUT2 (Figures 4e–h). Among the EM2-ir varicosities in contacting with FG-labeled PNs, the proportion of those were also VGLUT2-ir was 20.49 ± 5.27% in the SDH. Profiles that showed strong GAD67-immunoreactivity were numerous in lamina I–III. However, the varicosities containing both EM2 and GAD67 were not observed. The FG-labeled PNs in lamina I received contacts from EM2-ir or GAD67-ir profiles (Figures 4i–l).

### Ultrastructural features of endomorphin- 2-, substance P-, vesicular glutamate transporter 2- or GAD67-ir terminals and μ-opioid receptor-ir profiles and synaptic connections between them in the superficial spinal dorsal horn

**FIGURE 5:**
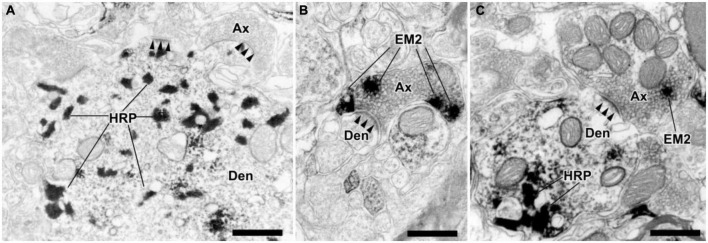
The ultrastructural features and synaptic connections between EM2-ir terminals and dendrites of the WGA-HRP retrogradely labeled neurons in lamina I of the spinal dorsal horn. **(A)** A dendritic profile (Den) of lamina I projection neurons contained TMB reaction products of the histochemistry for WGA-HRP. **(B)** A EM2-ir terminal (Ax) formed an asymmetrical synapse (solid arrowheads) with a immunonegative dendrite. **(C)** A EM2-ir terminal (labeled with DAB reaction products) made an asymmetrical synapse with a WGA-HRP-labeled dendritic profile. Scale bars = 500 nm **(A–C)**.

Under electron microscopy, the spinoparabrachial PNs containing WGA-HRP presented clumps of crystalline material with highly electron-dense in the cytoplasm and/or large dendrites (Figure 5A). The axonal terminals containing EM2 presented electron-dense DAB reaction products, which were homogeneously distributed. Neuropeptides usually located in large dense-coated granular vesicles (LDGV), which were observed in most of the EM2-ir terminals (Figures 5B–C). What is in accordance with our light microscope observing, the axonal terminals containing EM2-ir DAB reaction products formed synapses with the WGA-HRP-labeled dendritic profiles (Figure 5C). The majority of these synapses were asymmetrical, but symmetrical synapses were also found.

**FIGURE 6:**
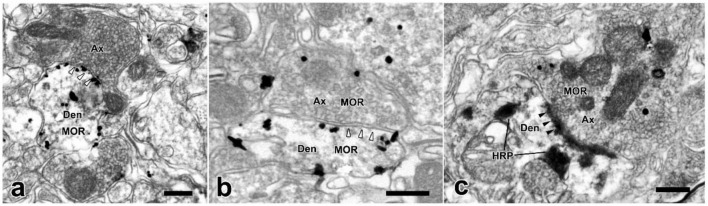
The ultrastructural features and synaptic connections between MOR-ir profiles and dendrites of the WGA-HRP retrogradely labeled neurons in lamina I of the spinal dorsal horn. **(a)** A immunonegative axon terminal (Ax) formed a symmetrical synapse (hollow arrowheads) with a MOR-ir dendrite (Den) (containing gold-silver grains). **(b)** A MOR-ir terminal (containing gold-silver grains) also formed a symmetrical synapse (hollow arrowheads) with a MOR-ir dendrite. **(c)** A MOR-ir terminal (Ax) formed an asymmetrical synapse (solid arrowheads) with a WGA-HRP labeled dendrite. Scale bars = 250 nm **(a–c)**.

**FIGURE 7:**
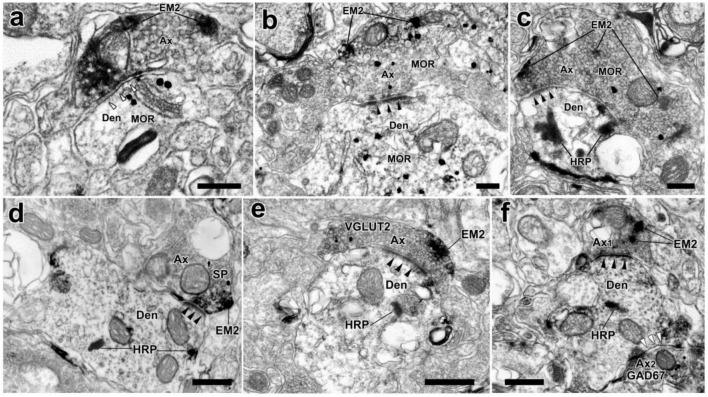
The synaptic connections between EM2-ir terminals and MOR-ir profiles, EM2/MOR-ir terminal, EM2/SP-ir terminal, or EM2/VGLUT2-ir terminal, or EM2-ir and GAD67-ir terminals and WGA-HRP retrogradely labeled dendrites in the spinal dorsal horn. **(a)** A EM2-ir terminal (Ax) labeled with DAB reaction products formed a symmetrical synapse (hollow arrowheads) with a MOR-ir dendrite (Den). **(b)** An asymmetrical synapse (solid arrowheads) was formed between an axon terminal containing both EM2 and MOR and a MOR-ir dendrite. **(c–e)** Axon terminals containing both EM2-ir DAB reaction products and immunogold-silver grains indicating MOR or SP or VGLUT2 formed asymmetrical synapses (solid arrowheads) with HRP-labeled dendrites indicated with TMB reaction products. **(f)** A EM2-containing terminal (Ax1) showed with DAB reaction products and a GAD67-containing terminal (Ax2) labeled with immunogold-silver grains made asymmetrical synapse (solid arrowheads) and symmetrical one (hollow arrowheads) with the same WGA-HRP labeled dendrite indicated with TMB reaction products, respectively. Scale bars = 250 nm **(a–c)**, 500 nm **(d–f)**.

MOR-ir were identified by the immunogold-silver grains, which located under the membranes of cell bodies, dendrites or in the cytoplasm (Figure 6a). Many MOR-ir immunogold-silver grains attached to the membranes of the synaptic vesicles were also observed in the axonal terminals (Figures 6b,c). Moreover, axon terminals filled with EM2-ir DAB reaction products and MOR-ir immunogold-silver particles formed asymmetrical synapses with WGA-HRP-retrogradely labeled neuronal dendritic processes in lamina I of the SDH (Figure 7c). Meanwhile, synaptic connections were detected between axon terminals filled with EM2-ir DAB reaction products and neuronal dendritic processes containing MOR-ir immunogold-silver particles (Figures 7a,b). SP-, VGLUT2-, and GAD67-ir profiles were also identified by the presence of the immunogold-silver particles in the axonal terminals. In accordance with the results observed from light microscope, axon terminals filled with both EM2-ir DAB reaction products and SP- or VGLUT2-ir immunogold-silver particles formed asymmetrical synapses with WGA-HRP-retrogradely labeled neuronal dendritic processes in lamina I of the SDH (Figures 7d,e). Whereas, axon terminal containing EM2-ir DAB reaction products and axon terminal containing GAD67-ir immunogold-silver particles formed asymmetrical synapse and symmetrical synapse with WGA-HRP-retrogradely labeled neuronal dendritic processes, respectively (Figure 7f).

## Discussion

In this study, we examined the contacts between endomorphin-2 (EM2)-ir terminals and projection neurons (PNs) in lamina I of the SDH projecting to the lateral parabrachial nucleus (LPB). Not only terminals containing EM2 but also terminals containing both EM2 and μ-opioid receptor (MOR) made synapses with PNs projecting the LPB, most of which were asymmetric synapses. Moreover, terminals containing both EM2- and substance P (SP)- or vesicular glutamate transporter 2 (VGLUT2)-ir also formed synapses with PNs. MOR-ir neurons in lamina II received contacts from EM2-ir varicosities, which was also confirmed by immunoelectron microscopy. All these results provided morphological evidence for the analgesic effects of EM2 through inhibiting activities of the spinoparabrachial PNs in the SDH.

### Distributions of projection neurons, endomorphin-2-ir, and μ-opioid receptor-ir profiles in the superficial spinal dorsal horn

PNs are mostly distributed in lamina I, scattered throughout lamina III-VI, and with very few present in lamina II in the lumbar spinal cord. As a major component of these PNs in the SDH, PNs in lamina I have been analyzed in detail through retrograde tracing studies (Spike et al., 2003; Todd, 2010). The lamina I PNs projected to the brain nucleus including the caudal ventrolateral medulla (CVLM), the nucleus of the solitary tract (NTS), the LPB, the periaqueductal grey (PAG) and certain nuclei in the thalamus (Todd et al., 2000; Spike et al., 2003; Todd, 2010). Although most PNs had only contralateral projections, some PNs projected bilaterally (Spike et al., 2003). Retrograde tracing studies show that PNs constituted ∼5% of lamina I neurons in the rat. Of all PNs in lamina I, 95% projected to the LPB, around a third to the PAG, a quarter to the NTS and <5% to the thalamus (Spike et al., 2003; Todd, 2010). There were extensive collateralizations of axons, with some PNs projecting to at least three nucleus (LPB, PAG and thalamus) (Spike et al., 2003; Al-Khater and Todd, 2009). Given the most PNs projecting to the LPB, we chose the PNs in lamina I projecting to the LPB as the investigators.

In the lumbar SDH, EM2-ir terminals originated from the ipsilateral dorsal root ganglion (DRG) neurons and bilateral hypothalamus and NTS (Hui et al., 2010; Zhu et al., 2011). Therefore, we could not deny the possibility that EM2-ir terminals originating from the NTS or hypothalamus contacted with the PNs in lamina I in this investigation. While the rats received both capsaicin *i.t.* injections and spinal transection at the 7th thoracic segment, EM2-ir fibers and terminals in the SDH were depleted completely (Hui et al., 2010). In this situation, we also did not observe EM2-ir cell bodies in the SDH. By using immunoelectron microscopy, the ultrastructures that EM2 was expressed in the inherent neurons in the SDH were not observed, either (Lu et al., 2022).

EM2 labeled MOR1 more potently than MOR2 like most opioids, which displayed a 5- to 10-fold greater affinity in the MOR1 binding assay (Goldberg et al., 1998). The high affinity of EM for MOR was also confirmed in competition studies against the cloned MOR1. Therefore, the antibody for MOR used in this investigation is against MOR1, which is also widely applied in the previous studies. Many MOR-ir neurons were observed in lamina II and a few in the dorsal part of lamina III. Very few MOR-ir cell bodies were seen in lamina I and no FG-labeled PNs containing MOR in our investigation, which was consistent with previous observations (Ding et al., 1996; Kemp et al., 1996; Liu et al., 2011). Moreover, if there were expressions of MOR on the PNs, applying EM2 will not only decrease the resting membrane potentials (RMP) but also increase the rheobase current (Wu et al., 1999; Fujita and Kumamoto, 2006; Chen et al., 2016). Our previous study show that applying EM2 to the PNs did not change both the RMP and rheobase current (Yin et al., 2018). These also suggest that the PNs in lamina I did not express MOR (Heinke et al., 2011). What is different, the majority of neurons projecting to the thalamus and LPB in lamina I of the trigeminal dorsal horn contained MOR-ir (Mitchell et al., 2004). There were fundamental differences in projections and MOR distributions between the trigeminal dorsal horn and lumbar spinal cord (Bereiter et al., 2000).

### Effects of endomorphin-2 on inhibitory synaptic transmission of projection neurons

Another difference seen between our investigation and other studies is that EM1, EM2, or other opioids could inhibit excitatory but not inhibitory information transmission in SG neurons (Yajiri and Huang, 2000; Koga et al., 2005). It has been shown that the MOR, δ-opioid receptor (DOR), and κ-opioid receptor (KOR) agonists had no effects on evoked or miniature inhibitory postsynaptic currents (IPSCs) mediated by either the GABA_A_ or glycine receptor (Kohno et al., 1999). However, DAMGO depressed the transmissions of glycine and GABA in SG neurons of the spinal trigeminal nucleus (Grudt and Henderson, 1998). There are indeed differences in opioid actions on the SG neurons between the spinal cord and spinal trigeminal nucleus. Our previous study has demonstrated that approximately 18.9% of MOR-ir neurons were GABA-ir in lamina II of the spinal trigeminal caudal nucleus (Li et al., 2014). But the great majority of the MOR-ir neurons (131 of 139; 94%) were not GABA-ir or glycine-ir in the SDH, which suggest that most of the neurons expressing MOR were excitatory interneurons (Kemp et al., 1996).

### Mechanisms of spinal analgesia produced by endomorphin-2

Cells containing EM2 are small- to medium-sized neurons in the DRG and these neurons also express SP, calcitonin gene related protein (CGRP), MOR, respectively, which matches the co-distributions of EM2/ SP-, EM2/ CGRP-, and EM2/MOR-ir varicosities in the SDH (Spike et al., 2002; Sanderson Nydahl et al., 2004; Luo et al., 2014; Wu et al., 2015). Particularly, the extensive co-distribution of EM2-ir and SP-ir in the DRG could be observed. Almost all EM2-ir neurons express SP (95.7%), and even higher percentage (99.1%) of the SP-positive neurons are EM2-ir (Sanderson Nydahl et al., 2004). Electron microscope study showed colocalization of EM2 and SP in the same large dense-cored vesicles of axon terminals in the superficial SDH, by using a post-embedding immunogold double-labeling protocol (Sanderson Nydahl et al., 2004; Wu et al., 2015). Meanwhile, there were MOR-ir gold particles located on both pre- and post-synaptic membranes. Intrathecal levels of SP were found to be decreased following *i.t.* injection of EM2, which is measured through *in vivo* microdialysis analysis of cerebrospinal fluid (Wu et al., 2015). These results suggest that EM2 is involved in controlling nociceptive information processing by modulating the releases of other neurotransmitters via pre-synaptic MOR. This very strong anatomical relationship between the co-expression of SP and EM2 underlined EM2 regulating the release of SP via MOR autoreceptors, which has been also observed in many other areas, such as NTS (Greenwell et al., 2007; Niu et al., 2009; Zhu et al., 2011). In our investigations, the majority of axon terminals containing MOR-ir formed asymmetric synapses with profiles belong to either HRP-labeled PNs or other neurons. In this situation, EM2 regulated the releases of presynaptic excitatory neurotransmitters (such as, glutamate, SP, and CGRP) by binding to the presynaptic MORs, which produced the inhibitory effects (Sanderson Nydahl et al., 2004; Greenwell et al., 2007; Wu et al., 2015; Gu et al., 2017).

DAMGO, the MOR agonist, reduced the amplitude of EPSCs in SG neurons which were monosynaptically evoked by stimulating Aδ afferent fibers and the frequency of miniature EPSCs without affecting their amplitude. The effect of DAMGO cannot be seen in the presence of MOR antagonist CTOP and DAMGO failed to affect the responses of SG neurons to bath-applied AMPA (Kohno et al., 1999). Evoked and miniature IPSCs were unaffected by DAMGO. Since the postsynaptic effects of MOR agonists on lamina II neurons were to induce hyperpolarization (Wu et al., 1999, 2003; Yajiri and Huang, 2000; Fujita and Kumamoto, 2006). The inhibition of excitatory interneurons (EINs), which were responsible for relaying nociceptive information to other neurons including the PNs, were involved in part of the analgesic action of opioids in the SDH.

**FIGURE 8:**
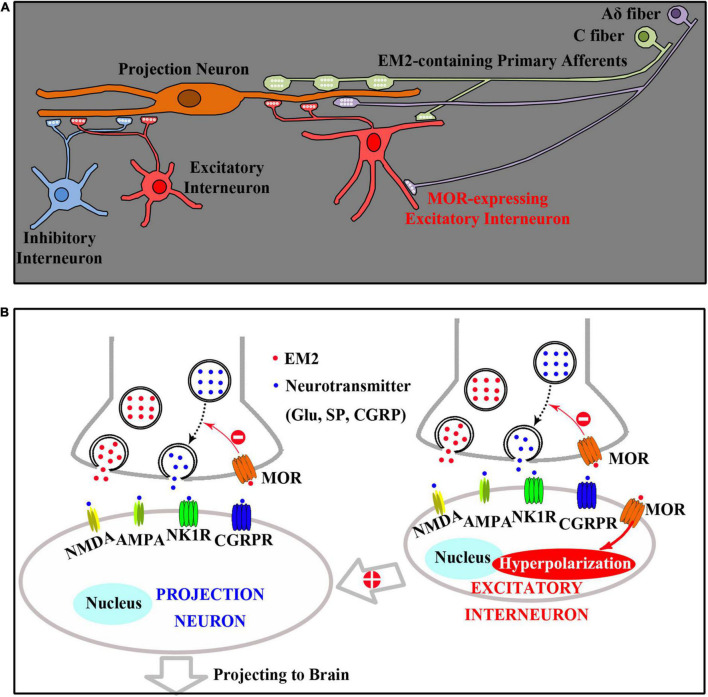
A diagram presenting the spinal analgesic mechanisms of EM2. **(A)** Both projection neurons (PNs) and excitatory interneurons (EINs) are innervated by EM2-containing primary afferents (C and Aδ fibers). **(B)** Primary afferent terminals releasing EM2 to act on presynaptic MOR of PNs reduces the release of excitatory neurotransmitters (such as glutamate, SP, and CGRP), which inhibits the activities of PNs in lamina I directly. Primary afferent terminals also releasing EM2 to act on both presynaptic and postsynaptic (Hyperpolarization) MORs of EINs, which inhibits the activities of PNs in lamina I indirectly by decreasing the excitatory inputs from EINs to PNs.

Combined the previous investigations with this study, the analgesic mechanisms of EM2 in the SDH includes two aspects, the direct and the indirect effects (Figure 8). Primary afferent terminals releasing EM2 to act on presynaptic MOR of PNs reduces the release of excitatory neurotransmitters (such as glutamate, SP, and CGRP), which inhibits the activities of PNs in lamina I directly. Primary afferent terminals also releasing EM2 to act on both presynaptic and postsynaptic MORs of EINs, which inhibits the activities of PNs in lamina I indirectly by decreasing the excitatory inputs from EINs to PNs.

## Data availability statement

The original contributions presented in this study are included in the article/supplementary material, further inquiries can be directed to the corresponding authors.

## Ethics statement

This animal study was reviewed and approved by the Committee of Animal Use for Research and Education of the Fourth Military Medical University.

## Author contributions

Y-QL and Y-LD conceived the project and designed the experiments. J-BY, TZ, FL, H-WG, JY, H-QH, and HL performed immunofluorescence staining. Y-CL, J-BY, TZ, TD, M-MZ, Z-ZK, TC, B-ZC, and Y-BC finished the electron microscope investigations. Y-QL, Y-LD, Y-CL, and J-BY wrote the manuscript. All authors contributed to the article and approved the submitted version.
